# Intra-abdominal Pressure Measurement as a Predictor of Postoperative Wound Complications in Patients Undergoing Emergency Laparotomy: A Prospective Observational Study

**DOI:** 10.7759/cureus.54860

**Published:** 2024-02-25

**Authors:** Dharmendra Dugar, Sunny Goel

**Affiliations:** 1 General Surgery, All India Institute of Medical Sciences, Raipur, IND; 2 General Surgery, University College Of Medical Sciences (UCMS) & Guru Teg Bahadur (GTB) Hospital, Delhi, IND

**Keywords:** surgical site infection, wound dehiscence, abdominal compartment syndrome, intra-abdominal hypertension, intra-abdominal pressure

## Abstract

Introduction

Elevated intra-abdominal pressure (IAP) hampers the effective functioning of intra- and extra-abdominal organs. Despite the abundance of knowledge, routine measurement of IAP still needs to be widely incorporated in managing at-risk patients. The present study intends to assess the need for IAP measurement on abdominal wound healing in emergency laparotomy patients.

Methods

This prospective study was carried out over 24 months in patients undergoing emergency laparotomy. The IAP was measured at admission, immediately after surgery, and during the early postoperative period at 6, 12, 24, 48, and 72 hours. The patients were evaluated for the development of wound-related complications over a follow-up period of three months post-operatively.

Results

Seventy-two patients were enrolled. At admission, 54 (75%) patients had intra-abdominal hypertension (IAH), of which three patients had evidence of abdominal compartment syndrome. Thirty-one (43%) patients developed postoperative wound infections. The overall incidence of wound infection was significantly higher in patients with IAH (54.3% vs. 24%, p-value = 0.04, Pearson's Chi-squared test). The frequency of wound dehiscence was greater (19.6 % vs. 4.3 %, p-value 0.14, Fischer's exact test) in patients with IAH. The median duration of hospital stay (13 vs. 8 days, p-value 0.02, Mann-Whitney U test) and healing time (30.5 vs. 18 days, p-value 0.02, Mann-Whitney U test) was significantly higher in patients with IAH.

Conclusion

Measurement of IAP is a relatively simple procedure that should be incorporated into the routine postoperative care of surgical patients. The presence of elevated IAP can identify the subset of patients at risk of increased postoperative wound complications.

## Introduction

Intra-abdominal pressure (IAP) is the pressure concealed within the abdominal cavity. The World Society of the Abdominal Compartment Syndrome (WSACS) has defined intra-abdominal hypertension (IAH) as a sustained or repeated pathological increase in IAP >12 mm Hg and abdominal compartment syndrome (ACS) as a severe form of IAH with a sustained IAP >20 mm Hg that is associated with the development of new organ dysfunction or failure [1]. Septic peritonitis, acute mesenteric ischemia, necrotizing pancreatitis, and traumatic or non-traumatic intra-abdominal hemorrhage are the conditions associated with an increased incidence of ACS [2]. The perils of IAH were identified more than two decades back when Sugrue et al. reported an 11-fold increase in mortality in the patients of IAH compared to those not having IAH [3]. Similarly, the mortality rate can reach 80-100% in the patients of untreated ACS [4]. An increase in IAP is also associated with an increased occurrence of wound dehiscence following laparotomies [5]. Abdominal wound dehiscence, in turn, is associated with an increased risk of nosocomial infection, peritoneal contamination, and incisional hernia. The WSACS recommendation suggests serial measurement of IAP at every four-hour interval in patients having ACS till the target IAP of <12 mm Hg is reached [1]. Despite the grave consequences of ACS, routine IAP measurement is still not a common practice, leading to large-scale underreporting of IAH and subsequent ACS [6]. Most of the work regarding IAH was carried out in critically ill patients in intensive care units (ICU) with round-the-clock monitoring facilities. The present study intends to assess the feasibility of routine IAP monitoring and the effect of IAH on abdominal wound healing in patients undergoing emergency laparotomy and managed postoperatively in non-ICU settings.

## Materials and methods

This prospective study was carried out in a single surgical unit of a tertiary care teaching hospital, University College of Medical Sciences and Guru Teg Bahadur Hospital (GTB) Hospital, Delhi, India, over 24 months. Consecutive adult patients (age ≥18 years) who underwent emergency laparotomy through a midline incision and consented to be part of the study were included. Patients with a history of prior abdominal surgery, failed attempt at Foley catheterization at admission, trauma to the urinary bladder, and in whom the abdomen was not closed intentionally for a second look laparotomy were excluded. Clearance was obtained from the institutional ethical committee for undertaking the study.

Detailed information regarding the patient's demographics (age, gender, and body mass index), hemodynamic status at admission, and clinical and biochemical evidence of organ failure was noted. The operative details (intra-operative findings, type of operative procedure, duration of surgery, placement of drains, and method of skin closure), postoperative diagnosis, and postoperative wound complications (infection, dehiscence, development of incisional hernia) were recorded.

Measurement of Intra-abdominal Pressure

Measurement of IAP can either be invasive or non-invasive. Invasive methods include a Veress needle during laparoscopy and peritoneal dialysis catheter in renal failure patients. The non-invasive means are a Foley catheter to monitor intravesical pressure, a nasogastric tube for intragastric pressure measurement, the central line to measure inferior vena cava pressure, manometry through a Jackson Pratt drain, and measuring rectal and uterine pressure. We preferred intra-vesicular pressure measurement as it was non-invasive, inexpensive, readily available, reliable, reproducible, and less cumbersome to perform in the emergency room. However, intravesical pressure measurement is fallacious in patients having bladder injury, pelvic fracture, bladder/pelvic hematoma, and neurogenic bladder. These patients were excluded from our study. Each patient was catheterized with a 16 Fr Foley catheter to empty the urinary bladder. Then, 50 ml of sterile saline was instilled into the bladder, and the catheter tubing was clamped. After one minute, intra-vesicular pressure was measured with a saline manometer at full expiration, with the patient lying in a supine position and relaxed abdominal muscles. Zero reference level was taken at the mid-axillary line level. A conversion factor of 1.36 was used to convert the measured pressure in centimeters into mm Hg.

Intra-abdominal pressure was measured soon after admission, after completion of the operation, and subsequently at an interval of six hours, 12 hours, 24 hours, 48 hours, and 72 hours in the postoperative period. The presence of IAH was categorized into four grades: grade 1 (IAP 12 - 15 mm Hg), grade 2 (IAP 16 - 20 mm Hg), grade 3 (IAP 21 - 25 mm Hg) and grade 4 (IAP > 25 mm Hg) as per WSACS guidelines [1]. The acute physiology and chronic health evaluation (APACHE) II score was calculated at the time of admission and at 72 hours after laparotomy in all the patients. The abdominal wound complications were identified according to guidelines recommended by the Centre for Disease Control and Prevention (CDC) for diagnosis of surgical site infections [7]. Wound infection or superficial surgical site infection was considered when the depth of infection was confined to the skin, and subcutaneous tissue and wound dehiscence were defined as the separation of the abdominal musculoaponeurotic layers. Complete epithelisation in open wounds and the absence of signs of dehiscence after suture removal in sutured wounds was considered as adequate wound healing.

**Figure 1:**
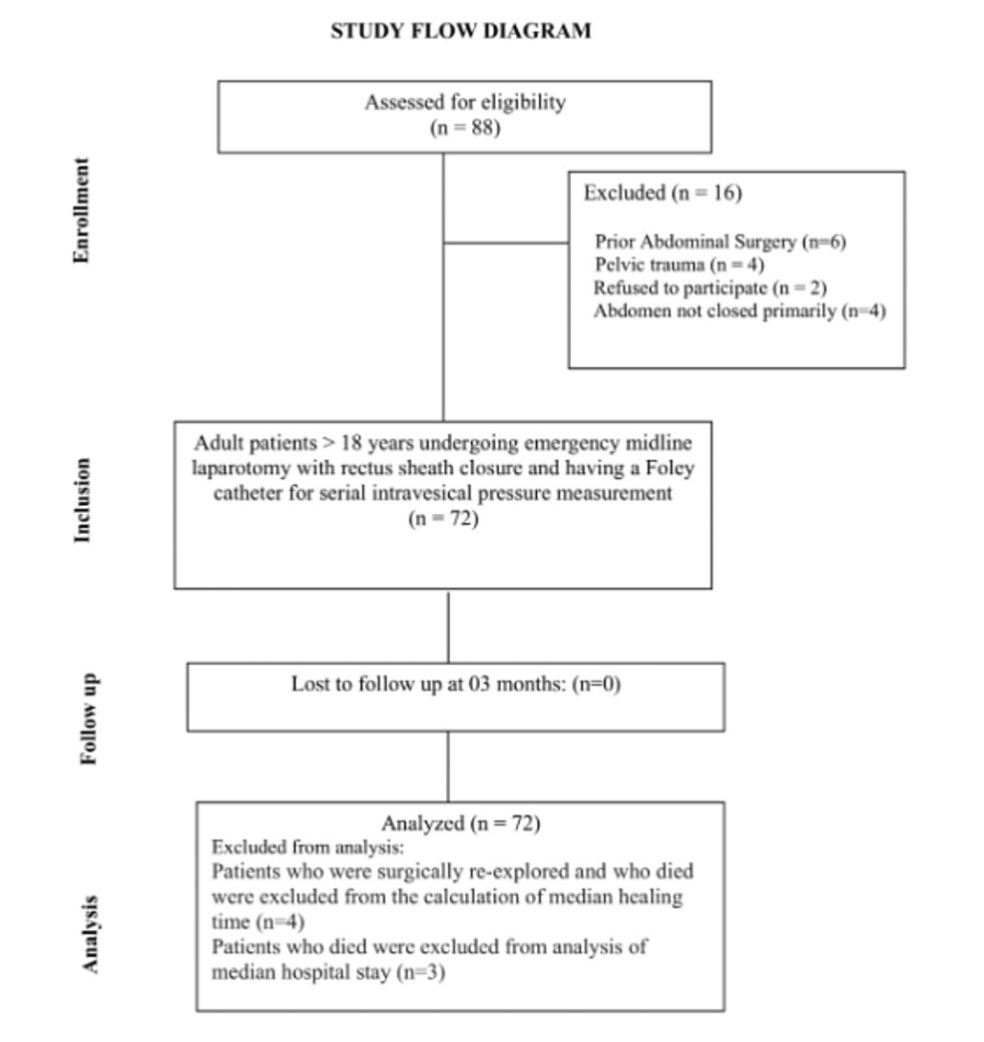
Flow of the study

The patients were discharged when their oral feeding was sufficient to meet daily nutritional requirements, absence of fever for 48 hours, and a healing abdominal wound that could be managed on outpatient visits. The patients were called for follow-up at two weekly intervals in the first month and then monthly for three months (Figure 1). 

## Results

Data pertaining to the 72 (53 male and 19 female) enrolled patients were analyzed. The mean age of the patients was 33.2 ± 13.7 years (range 18-65 years), and the mean BMI was 19.4 ± 3.8 kg/mt2 (range 12.3- 31.2 kg/mt2). The mean APACHE II score at admission was 6.2 ± 5.9 (range 0-31). Fifty-one (70.8%) patients were operated within 12 hours of admission. The most common indication for laparotomy was hollow viscous perforation (n = 50, 69%), followed by intestinal obstruction (n = 12, 17%). In 37 (51.4%) patients, the volume of peritoneal collection at laparotomy was ≤500 ml, and in the remaining 35 (48.6%) patients, the volume was >500 ml. The mean duration of surgery was 141.5 ± 70.3 min (ranged 50 - 360 min). The duration of surgery was less than two hours in 39 (54.2%) patients. The mean IAP of the 72 patients at various time intervals was measured (Table 1).

**Table 1:** Mean intra-abdominal pressures in 72 patients * The data does not include patients who died: one patient between 24 - 48 hour and one patient between 48 - 72 hour IAP - intra-abdominal pressure

Time	Mean IAP (mm Hg)	Standard deviation	Range (mm Hg)
Post admission (PA_0_)	14.4	4.4	3.7 - 22.8
0-hour postoperative (PO_0_)	15.8	5.5	7.4 - 37.5
6-hour postoperative (PO_6_)	14.4	5.2	3.7 - 30.9
12-hour postoperative (PO_12_)	14.0	4.4	3.7 - 23.5
24-hour postoperative (PO_24_)	12.9	4.6	3.7 - 25.7
48-hour postoperative (PO_48_)*	11.6	4.4	2.9 - 23.5
72-hour postoperative (PO_72_)*	10.9	4.3	2.9 - 23.5

**Figure 2:**
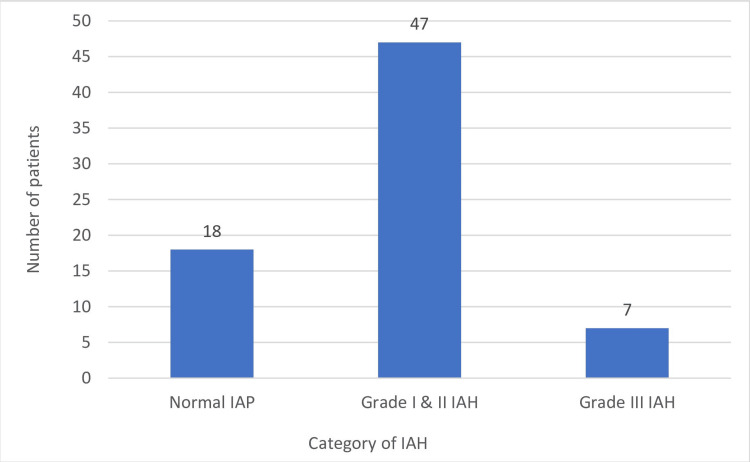
IAH at the time of admission IAH - intra-abdominal hypertension

The mean IAP was found to be greatest when measured soon after the operation. At admission, IAH was diagnosed in 54 (75%) patients, grade I and II IAH in 47 (65.3 %), and grade III IAH in seven (9.7%) patients (Figure 2). Immediate improvement in IAH following operative intervention was observed in 12 (22.2%) patients, while the rest, 42 (77.8%), continued to have IAH for a variable period of time in the early postoperative period. Additionally, five patients with normal IAP at admission developed IAH following operative intervention, making a total of 47 (65.3%) patients with IAH in the postoperative period. The presence of IAH was found to have a significant association with the mean APACHE II score at admission (6.8±5.2 vs. 3.7±4.7, p-value = 0.01, Student t-test) and duration of surgery of >2 hours (26 patients versus seven patients, p-value = 0.04, Fisher exact test). The volume of peritoneal collection at laparotomy of > 500 ml was also found to be significantly associated with the level of IAP (15.7±3.5 mm Hg vs. 13.1±4.7 mm Hg, p-value = 0.01, Student t-test).

The rectus sheath closure was performed in all the patients at the index surgery. The skin was primarily closed in 38 (52.8%) patients, while delayed primary closure (DPC) was attempted in 21 (29.2%) patients between the third and 10th postoperative days. In the remaining 13 (18%) patients, the abdominal wound was allowed to heal with secondary intention due to persistent infection. Thirty-one (43.1%) patients had evidence of wound infection in the postoperative period. The overall incidence of wound infection in patients with IAH was significantly more than in patients with normal IAP (54.3% vs. 24%, p-value = 0.04, Pearson's Chi-squared). Attempt at primary closure of skin was associated with an increased incidence of wound infection in patients with IAH in comparison to patients having normal IAP, though the difference was not statistically significant (50% vs. 33.3%, p-value > 0.05, Fischer exact test). A total of 10 (13.9%) patients (one having normal IAP and nine patients with IAH) developed wound dehiscence involving disruption of fascio-muscular layer in the postoperative period. In six patients, the dehiscence was detected in ≤7 days, while it was evident at >7 days in four patients. The incidence of wound dehiscence was higher in patients with IAH than in patients with normal IAP (16.66 % vs. 5.55 %); however, the difference was not statistically significant (p-value > 0.05, Fischer exact test).

Seven (70%) patients of wound dehiscence were subsequently detected to have an incisional hernia at the end of three months follow-up. All three patients having ACS improved following surgical intervention. Three (4.2%) patients in our study died as a result of uncontrolled sepsis and multi-organ failure. The median duration of hospital stay of our patients who survived was 11 ± 11 days. The median healing time in patients who survived and were not subjected to re-exploration was 23 ± 52.5 days. The various outcome measures were compared between patients with normal IAP and patients with IAH and were presented in tabular form (Table 2).

**Table 2:** Outcome in patients with normal IAP and patients with IAH *Patients who were surgically re-explored and who died were excluded from the calculation of median healing time **Patients who died were excluded from analysis of median hospital stay IAP - intra-abdominal pressure; IAH - intra-abdominal hypertension; ACS - abdominal compartment syndrome

Outcome Measures	Normal IAP (n = 25)	IAH and ACS (n = 47)	p-value
Patient with organ failure at admission	2 (8.0%)	7 (14.9%)	0.48 (Fischer's exact test)
Patients with wound infection	6 (24%)	25 (54.3%)	0.04 (Pearson Chi-squared)
Patients with wound dehiscence	1 (4.3%)	9 (19.6%)	0.14 (Fischer's exact test)
Patients with incisional hernia	0 (0%)	7 (15.2%)	0.08 (Fischer's exact test)
Median healing time (days)*	18 ± 14.5	30.5 ± 62.5	0.02 (Mann-Whitney U test)
Median hospital stays (days)**	8 ± 3.5	13 ± 11.5	0.03 (Mann-Whitney U test)
Mortality	2 (8.0%)	1 (2.1%)	0.27 (Fischer's exact test)

## Discussion

Uneventful abdominal wound healing has been of prime importance in patients undergoing emergency laparotomy. Wound infection, wound dehiscence, and subsequent delayed wound healing result in prolonged hospitalization, increasing the burden on hospital resources. The predisposing factors for impaired wound healing are advanced age, anemia, hypoalbuminemia, diabetes mellitus, malignancy, jaundice, obesity, malnutrition, intra-abdominal sepsis, poor wound closure technique, ionizing radiation, hypoxia, corticosteroids [8]. The detrimental effects of IAH and ACS on postoperative patient outcomes have been increasingly recognized worldwide. Still, routine measurement of IAP in perioperative settings has not yet been incorporated into the standard management protocol in many institutions. A study in the UK revealed that the majority (93%) of respondents measured IAP only in patients suspected of being at risk of developing ACS [6]. Uncertainty over the clinical utility of IAP measurement, IAP measurement technique, and interpretation of the measurement data contributed to the lack of enthusiasm among health professionals for rigorous IAP measurement in critical patients. The recent WSACS guidelines recommend IAP monitoring in all patients with abdominopelvic injury and disease requiring early surgical or radiological intervention [1]. Serial clinical evaluation is a poor predictor for identifying patients with IAH and is not preferable to traditional intravesical pressure measurement [9]. Various minimally invasive techniques, apart from conventional IAP monitoring methods, such as strain gauge, respiratory inductance plethysmography, bioelectrical impedance analysis, microwave reflectometry, and ultrasound with Doppler for abdominal wall thickness and tonometry are being explored, each with varying success rates [10]. 

The clinical significances of IAP monitoring are manifold, such as assessment of the effect of treatment with better resuscitation end points, early recognition of the potential life-threatening poly-compartment syndrome like cardio-abdominal-renal syndrome, and timely intervention to prevent ACS. Routine IAP monitoring is invaluable in reducing the morbidity and mortality of patients undergoing emergency surgery. Postoperative IAP levels are also more closely related to the development of wound complications, thus guiding resuscitation and management algorithms. Our study reaffirms the increased incidence of postoperative wound infection in patients with IAH. A raised IAP adversely affects blood flow to the abdominal wall, leading to ischemia of the muscles and fascial layers [5]. Consequently, there is an increased risk of wound infection and subsequent wound dehiscence in patients with IAH. In our study, most of the patients with elevated IAP at admission were also found to have IAH in the early postoperative period. Thus, even a single measurement of elevated IAP pre-operatively can help identify patients at high risk of postoperative wound infection. The various risk factors implicated in the development of IAH are decreased abdominal wall compliance, increased intraluminal contents, increased intra-abdominal contents, capillary leak, and overzealous resuscitation [1]. Definitive surgical intervention coupled with supportive measures such as nasogastric decompression and intra-peritoneal drainage catheters often result in rapid correction of intraluminal or intra-abdominal risk factors. The incidence of wound dehiscence, organ failure, and mortality rate was higher in patients with IAH; however, a significant association between them could not be established (p-value > 0.05). The mean pre- and postoperative IAP values were positively correlated with hospital stay and healing time due to the increased incidence of wound infection and wound dehiscence in patients with IAH.

The wound closure technique was also extensively studied as one of the factors responsible for postoperative wound infection. Delayed primary skin closure is considered a better option in wound closure for patients undergoing emergency laparotomy [11]. In this study, patients with primary skin closure had a higher incidence of wound infection and wound dehiscence than patients with IAH who underwent delayed primary skin closure.

A limitation of the present study is the small sample size, which may have prevented the establishment of significant differences in outcomes for several factors. The results of the present study need further confirmation by another study with a larger sample size. The present study comprises only patients requiring emergency laparotomy, so the results are not generalizable to the spectrum of patients undergoing elective laparotomies. Further studies are also essential to determine the role of temporary abdominal wound closure, vacuum-assisted closure techniques, and using prosthetic mesh devices or Bogota bags in reducing the frequency of IAH and ACS in the postoperative period [12,13]. The study protocol also deviated from the recommended guidelines by the WSACS, such as intravesical instillation of 50 ml of sterile saline rather than the instillation volume of 25 ml and a longer interval of 6 to 24 hours between consecutive IAP measurements. The effect of nasogastric aspiration and intraperitoneal drainage catheter on IAH and postoperative wound complications was also not separately evaluated in this study.

## Conclusions

The measurement of IAP is a simple and inexpensive procedure, feasible in almost all patients as a part of routine postoperative care. Documentation of IAH at admission should alert the surgeon to the possibility of encountering increased postoperative wound complications. This study highlights the significance of serial IAP measurements in patients scheduled for emergency laparotomy. Prompt implementation of corrective measures - such as ensuring adequate analgesia and sedation, nasogastric decompression, balanced fluid resuscitation, and early vital organ support, can mitigate the local and systemic adverse effects of IAH and ACS.
